# Assessing Species Fractional Cover and α‐Diversity in Boreal Peatlands Across Trophic Levels Using Hyperspectral Data

**DOI:** 10.1002/ece3.71941

**Published:** 2025-08-08

**Authors:** Sini‐Selina Salko, Aarne Hovi, Iuliia Burdun, Jussi Juola, Susanna Karlqvist, Miina Rautiainen

**Affiliations:** ^1^ Aalto University School of Engineering Espoo Finland

**Keywords:** close‐range sensing, fractional cover, peatland, reflectance spectra, spectral library, vegetation mapping

## Abstract

Boreal peatlands, which act as significant sinks and storage of global soil organic carbon, are increasingly threatened by the changing climate conditions as well as land use changes. Despite the importance of these ecosystems, their vegetation and ecological features remain poorly mapped compared to other terrestrial ecosystems. Hyperspectral satellite imaging shows promise for detailed vegetation mapping and biodiversity monitoring of boreal peatlands. However, its effective application requires a fundamental understanding of the spectral properties of the vegetation communities of boreal peatlands. To address this, we combined newly available, open‐source data consisting of close‐range sensed spectral libraries of boreal peatland vegetation communities and single species. Our aim was to examine the extent to which close‐range spectral data can be used to predict species‐specific fractional cover in minerotrophic and ombrotrophic peatland habitats using hyperspectral and multispectral data, and to assess the connection between spectral signatures and α‐diversity of the vegetation communities. Our findings show that hyperspectral data can be used to predict the fractional cover of certain plant species with moderate accuracy (*R* ^2^ = 0.58). When comparing data types, hyperspectral data typically produced slightly better model fits for species with larger sample sizes, appearing to be superior to multispectral data. However, in certain cases, such as in the prediction of litter cover in ombrotrophic peatland habitats, multispectral data yielded marginally better results (*R* ^2^ = 0.4–0.45). Furthermore, using hyperspectral data, we observed that the prediction of α‐diversity of the ombrotrophic habitats was moderately better (*R* ^2^ = 0.44) than that of the minerotrophic habitats (*R* ^2^ = 0.22). These results enhance our understanding of the spectral properties of the complex, multilayered vegetation communities and thus aid in the mapping of these vital ecosystems.

## Graphical abstract

The reflectance spectra of minerotrophic and ombrotrophic peatland vegetation communities vary notably.

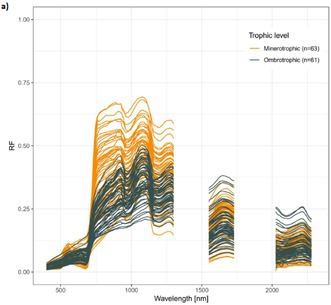

## 1 Introduction

Northern peatlands are one of the largest stores of soil organic carbon, and throughout the Holocene, they have served as significant sinks and storage for organic carbon (Nichols and Peteet 2019; Yu et al. 2010). However, anthropogenic warming and land use changes have been observed to alter the functioning of boreal peatland ecosystems, threatening to turn them into net sources of atmospheric carbon (Hugelius et al. 2020; Loisel et al. 2021; Nordström et al. 2022). Land use change‐related drivers of peatland degradation vary regionally, with agriculture playing a major role in North America, forestry drainage and peat extraction in northern Europe (Fluet‐Chouinard et al. 2023), and oil sand mining affecting areas in Canada and Siberia to some extent (Engering et al. 2022; Rooney et al. 2012). At the same time, changing climate conditions have been observed to alter these habitats in terms of biodiversity (Christiani et al. 2025) and carbon balance (e.g., Helbig et al. 2022; Zhang et al. 2022), impacts which are expected to continue (Loisel et al. 2021; Helbig et al. 2020; Hugelius et al. 2020). Given the substantial warming potential of peatland ecosystems' carbon emissions, accurate assessment and monitoring of peatland ecosystems and their associated changes are essential for understanding the global carbon cycle. The heterogeneity (Räsänen and Virtanen 2019) and difficult access (Minasny et al. 2019; Joosten and Clarke 2002) of these ecosystems pose challenges in monitoring them in the field, and thus, remote sensing techniques may provide tools for their detailed, spatially and temporally comprehensive mapping. However, much of the current research is limited to a small number of plant species (Lees et al. 2019; Girard et al. 2020) or research sites (Räsänen et al. 2019; McPartland et al. 2019) and often encompasses only the spectral regions of visible light and near‐infrared, excluding the shortwave‐infrared regions (Pang et al. 2022; Kolari et al. 2022).

Surface vegetation of boreal peatlands is used as an established indicator of the ecosystem's status, both in classifying the site's ecohydrology as well as studying peatland disturbances (Camill et al. 2009; Rydin and Jeglum 2006). Ecohydrology, which refers to the temporal and qualitative variation in the incoming water of a site, is strongly interconnected with peatland vegetation (Rydin and Jeglum 2006). The ecohydrology of a site is expressed with so‐called trophic levels, distinguishing sites that receive water solely from precipitation (ombrotrophic) from those that receive water from both precipitation and other sources, such as groundwater or surface water (minerotrophic) (Rydin and Jeglum 2006). During the growing season, the surface vegetation of a minerotrophic site is typically characterized by annual vascular plants. In contrast, an ombrotrophic site's vegetation is often mostly covered by bryophytes, with fewer vascular plants (Laine and Vasander 1996). The species diversity of the vegetation can be expressed with the term alpha‐diversity (α‐diversity) (Whittaker 1972), which refers to diversity on a local scale, describing species richness within a functional community (Andermann et al. 2022). In peatland vegetation communities, α‐diversity serves as an indicator of the site's nutrient status within the trophic level. Specifically, a higher α‐diversity value indicates higher nutrient availability across both ombrotrophic and minerotrophic site types.

Airborne and close‐range hyperspectral remote sensing have been successfully applied to retrieve information regarding the vegetation conditions as an indicator of the ecosystem status of different peatland ecosystems (Lopatin et al. 2019; Pang et al. 2024; Stuart et al. 2022). Hyperspectral satellite imaging, a novel and powerful method in vegetation mapping, offers a promising approach to monitoring the peatland vegetation communities' biodiversity, seasonality, and responses to changing climate conditions (Schweiger and Laliberté 2022). Hyperspectral mapping has shown promising results in monitoring both plant species and biodiversity in a range of different ecosystems (Davidson et al. 2016; Schweiger and Laliberté 2022; Wallis et al. 2024; Yang et al. 2023), including temperate peatlands (Arasumani et al. 2023; Mohammedshum and Kooistra 2015; Simpson et al. 2024). Exploring the spectral properties of boreal peatlands enhances our understanding of peatland ecosystems by complementing existing research on temperate and tropical peatlands, thereby addressing important knowledge gaps in remote sensing of these ecosystems. Spectral properties can be explored using species‐specific spectral libraries that include both the more extensively studied plant functional types (Pang et al. 2022; Räsänen, Aurela, et al. 2020; McPartland et al. 2019) and bryophyte species (Ito et al. 2025; Wolff et al. 2024; Salko et al. 2023b) as well as the less studied vascular plant species. This study seeks to facilitate the effective use of species‐specific hyperspectral satellite data for peatland monitoring by providing a fundamental understanding of the spectral properties of peatland vegetation communities.

To create this understanding and explore the possibilities and limitations of the use of hyperspectral data, close‐range spectral libraries of boreal peatland vegetation must first be measured. To address the potential of using spectral data in mapping the species and biodiversity of boreal peatland vegetation, we measured and analyzed new, open‐source spectral libraries of boreal peatland vegetation communities and species. The objective of this study was to assess the extent to which spectral close‐range hyper‐ and multispectral data can be used to predict the fractional cover of species in peatland vegetation communities across both minero‐ and ombrotrophic habitats, as well as to investigate the extent to which the spectral signature of a peatland vegetation community is connected to its α‐diversity.

## 2 Materials and Methods

### 2.1 Overview of the Study Sites and Measurements

This study is based on 124 vegetation plots measured in Estonia and Finland during the growing seasons of 2022 and 2023. These vegetation plots represent two different trophic levels: minerotrophic (63 plots) and ombrotrophic (61 plots). The differentiation between the two trophic levels was done during the field measurement by assessing the peatland type of the entire growing environment (Rydin and Jeglum 2006): firstly, using the tree cover as an indicator of the site's nutrient availability, and then, using the species of the ground vegetation. The site type division, an established way to study boreal peatland vegetation, has been shown to be useful for remote sensing applications (Isoaho et al. 2024; McPartland et al. 2019; Middleton et al. 2012).

**FIGURE 1:**
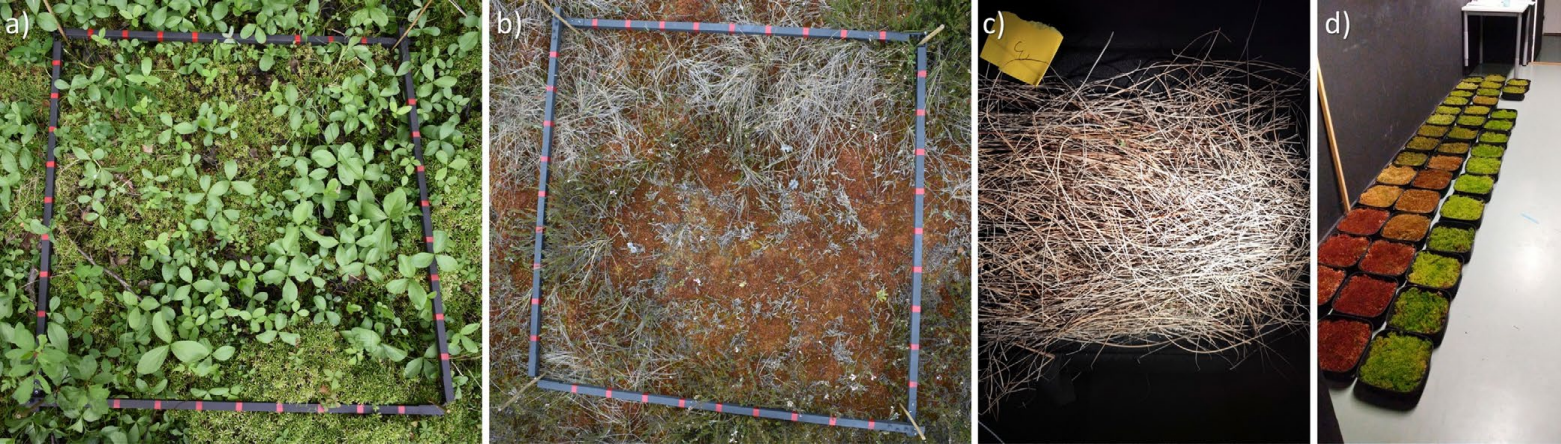
Example photographs of (a) a minerotrophic vegetation plot, (b) an ombrotrophic vegetation plot, (c) a litter sample, and (d) a selection of samples of the *Sphagnum* moss species. Photo credit: Iuliia Burdun (a and b), Sini‐Selina Salko (c and d).

In the plots, we conducted spectral measurements (Section 2.1.1) and estimated the fractional cover of different plant species (Section 2.1.4). In addition, we measured the leaf‐level spectral properties of dominant vascular plant species and litter in laboratory conditions (Section 2.1.2). Our measurements were complemented by previously published spectral libraries on, e.g., common moss and dwarf shrub species (Section 2.1.2). These species‐specific spectra were used to compose an endmember spectral library for a spectral unmixing analysis. All datasets used in this research are publicly accessible with their digital object identifiers (DOIs) listed in Table 1. Example photographs of vegetation plots and samples measured in the laboratory are provided in Figure 1.

**TABLE 1:** Open‐source spectral libraries used in the study.

Name	Digital object identifier	Species
Vegetation plots	https://doi.org/10.17632/3866tj3w8v.1	
*Sphagnum* mosses	https://doi.org/10.17632/wm5fcxdmzd.3	*Sphagnum angustifolium* (C.E.O.Jensen ex Russow) C.E.O.Jensen
		*Sphagnum capillifolium* (H.Klinggr.) H.Klinggr.
		*Sphagnum centrale* C.E.O.Jensen
		*Sphagnum cuspidatum* Ehrh. ex Hoffm.
		*Sphagnum fallax* (H.Klinggr.) H.Klinggr.
		*Sphagnum fuscum* (Schimp.) H.Klinggr.
		*Sphagnum rubellum* (Schimp.) H.Klinggr.
Litter	https://doi.org/10.17632/kg7627wgp6.1	Dead *Carex* sp. L.
Vascular plants	https://doi.org/10.17632/kg7627wgp6.1	*Betula nana* L.
		*Calla palustris* L.
		*Chamaedaphne calyculata* (L.) Moench
		*Comarum palustre* L.
		*Filipendula ulmaria* (L.) Maxim.
		*Menyanthes trifoliata* L.
		*Phalaroides arundinacea* (L.) Rauschert
		*Ranunculus repens* L.
		*Rubus chamaemorus* L.
		*Salix* sp. L.
		*Vaccinium uliginosum* L.
		*Viola palustris* L.
Dwarf shrubs	https://doi.org/10.14214/sf.22014	*Calluna vulgaris* (L.) Hull
		*Vaccinium myrtillus* L.
		*Vaccinium vitis‐idaea* L.

#### 2.1.1 Measurements and Preprocessing of the Vegetation Plot Spectra

The spectral measurements of the vegetation plots were conducted in Finland and Estonia in undrained peatlands in the national parks. During the growing season of 2022, measurements were carried out in Kurjenrahka (13.6–17.6 and 4.7–8.7), Teijo (6.6–10.6), Torronsuo (11.7–20.7) and Valkmusa (30.5–3.6). During the growing season of 2023, they were conducted in Luhasoo (1.6–3.6), Männikjärv (25.5–27.5), Patvinsuo (27.6–30.6), Soomaa (22.5–24.5) and Tammeluha (29.5–30.5). Each of these peatland sites is located either in the hemi‐boreal or boreal vegetation zone. The measurements have been described in detail by Salko, Hovi, Burdun, et al. (2024b) and are summarized below.

The spectral measurements were performed by placing a 1 m × 1 m sampling frame in a west‐to‐east direction over undisturbed peatland vegetation. Reflectance factors were recorded (350–2500 nm) with a FieldSpec 4 Standard‐Res spectrometer (serial number: 18456 in 2022 and 18,641 in 2023, manufacturer: Analytical Spectral Devices (ASD)) using a bare fiber optic cable. Each measurement was taken above the center of the sampling frame from approximately 85 cm height, with a field of view of 25°, resulting in a circular area viewed by the spectrometer of approximately 40 cm in diameter. The measurements were conducted under diffuse illumination conditions, minimizing the effect of solar zenith angle and thus ensuring that the spectral data collected at different latitudes were comparable to each other. A set of spectral measurements consisted of (1) a measurement of white reference spectrum using a calibrated Spectralon panel with 99% nominal reflectance, (2) a measurement of the target, and (3) a dark current measurement repeated once per optimization. Depending on the illumination conditions, the instrument was optimized after every plot, every two plots, or at most every three plots. Each of these measurements comprised 15 spectrometer readings averaged into one. The white reference measurement, the target measurement, and the dark current measurement were used to calculate the hemispherical‐conical reflectance factor (HCRF) (Schaepman‐Strub et al. 2006) with the method described in (Salko, Hovi, Burdun, et al. 2024b).

After the preprocessing, the HCRF spectra were divided into three data quality classes based on the level of random noise. For this study, we only used the best quality data (i.e., the category with noise free data at all wavelengths outside the atmospheric water absorption regions). The plots were then further filtered based on vegetation. From all measured plots at each peatland site, we selected only those where the vegetation consisted entirely of species represented in the species‐specific spectral libraries (Table 1). This filtering resulted in a final dataset of 124 plots. For these plots, we then calculated the fractional cover of each species (Section 2.1.4, Table 3).

#### 2.1.2 Measurements and Preprocessing of the Species‐Specific Spectra

We acquired the endmember spectra for dominant peatland plant species from two main sources: (1) new laboratory measurements of leaf‐level spectral properties of dominant vascular plant species and litter, and (2) established spectral libraries from previous studies on common peatland species. Below, we first detail our new measurements and then outline the additional spectral libraries utilized.

We measured the leaf‐level spectra of a total of 12 dominant vascular plant species in laboratory conditions. First, the samples were retrieved from their growing environment by carefully dislodging the whole plant and placing it in a cool container. To avoid taking all the samples from the same growth, each plant was collected at a minimum distance of 10 m from the other sampled plant individuals. After collection, the plant was transported indoors and kept in a cool container or fridge. The samples were measured within 2–6 h after the collection. A leaf was separated from the plant just before the measurement to avoid drying of the leaf tissue. For each species, nine separate leaves were measured, so that a maximum of three leaves were taken from one plant individual. In other words, from a plant with several leaves per stem, such as *Filipendula ulmaria* , three different leaf samples were detached from one stem, whereas, for a plant with one leaf per stem, such as *Rubus chamaemorus* , that leaf was used as the sample. The leaves were measured from both adaxial (upper) and abaxial (lower) sides. The spectral measurements were conducted with a spectrometer (ASD FieldSpec 4 Standard‐Res, serial number 18456 in 2022, 18,641 in 2023) to which a double integrating sphere (SpectroClip‐TR; Ocean Optics) was attached. The SpectroClip‐TR had two integrating spheres with 20 mm diameter and as an illumination source, it used a 20 W Tungsten halogen lamp (HL‐2000‐HP‐FHSA; Ocean Optics). During the measurement, the leaf was positioned within a 6 mm diameter sample port between the integrating spheres, and the clip was closed. The method is described in detail by Mõttus et al. (2017). The sampled plant species were selected based on the size of the leaf, that is, the leaf had to cover the entire sample port of SpectroClip‐TR. Reference measurements (white reference from a Spectralon panel with 99% nominal reflectance, dark current, as well as measurements of empty reflectance and transmittance spheres) were conducted at the beginning of the measurement session as well as between every third target measurement. Each measurement (target or reference) comprised of eight spectrometer readings averaged into one. To calculate the directional‐hemispherical reflectance factor (DHRF) spectra, the data were processed using the algorithm described in (Mõttus et al. 2017).

The spectral measurements of litter, consisting mainly of dead *Carex* sp., were conducted in a dark spectroscopy laboratory located at Aalto University, Espoo, Finland. A total of 12 litter samples of approximately the size of 20 cm × 20 cm were obtained from an undrained peatland site located in Vaipo, Kirkkonummi in autumn 2023, before the beginning of the snow‐covered season. The samples were transported to the spectroscopy laboratory, carefully positioned on a black tray, and measured immediately after transportation with a spectrometer (ASD FieldSpec 4, serial number: 18641). The reflectance spectrum at 350–2500 nm was recorded with the spectrometer using the bare fiber optic cable. The sample was positioned 30 cm below the nadir‐viewing fiber optic cable and illuminated by a 50 W Quartz Tungsten Halogen lamp with a beam that had an opening angle of 36° and an illumination‐restricting aluminum shade. With the 25° field of view, the recorded area was ~13.4 cm in diameter. Here, a set of spectral measurements consisted of (1) a white reference spectrum using a calibrated Spectralon panel (99% nominal reflectance), (2) a target measurement, and (3) a dark current measurement. The white reference and the dark current measurements were repeated at the beginning, end, and every 1 h from the start of the measurement session. Each measurement comprised 15 spectrometer readings averaged into one, and the calculation of the conical–conical reflectance factor (CCRF) (Schaepman‐Strub et al. 2006) was done by applying the method described in (Salko et al. 2023b).

For vascular plant species with leaves too small to measure with the SpectroClip‐TR (e.g., *Calluna vulgaris* ) or those that typically exhibit substantial stem cover compared to leaf cover (e.g., *Vaccinium myrtillus* ), we used spectral measurements of pure single‐species canopies performed outdoors. The spectral measurements were taken with a spectrometer (ASD FieldSpec 4 spectrometer, serial number 18456) under diffuse illumination conditions, from 15 to 35 cm above the vegetation sample with the field of view of 25°. This resulted in a circular area viewed by the spectrometer of approximately 6.6–15.6 cm in diameter. A total of three vegetation samples per species were measured. The HCRF was obtained by using the target measurement, the averaged white reference spectral measurement that was conducted before and after the target measurement, and the dark current measurement. For a more detailed description of the procedure, refer to Kuusinen et al. (2023).

For the bryophyte species, consisting of *Sphagnum* mosses, the measurements were conducted with the same measurement protocol as used for litter. The 21.5 cm × 21.7 cm samples were collected from four undrained peatland sites in southern Finland and measured in the spectroscopy laboratory with a spectrometer (ASD FieldSpec 4, serial number 18456) within 2–6 h after transportation. Ten samples were measured per species. Similarly to the litter measurements, the CCRF was obtained by using the averaged white reference spectra, target measurement, and the dark current measurement. For a detailed description of the procedure, refer to Salko et al. (2023b).

#### 2.1.3 Harmonization of the Spectral Data

The vegetation plot spectra and the endmember spectra were harmonized by first applying the Savitzky–Golay smoothing algorithm with second order polynomial and a window size of 15 nm in the 350–1000 nm region, 39 nm in the 1001–2000 nm region, and 75 nm in the 2001–2500 nm region on all the data. We then removed the atmospheric water absorption regions at 1330–1549, 1761–2024, and 2311–2500 nm because of the high level of noise present in the data in these regions. Also, the spectral region 350–399 nm was removed due to the high noise level in these wavelengths in the measurements conducted with the integrating sphere. As a result, the spectra from three distinct wavelength regions were used in the analyses: 400–1329, 1550–1760, and 2025–2310 nm. The first region covers the visible (VIS, 400–700 nm) and near‐infrared (NIR, 700–1300 nm) regions, and the latter two cover the shortwave‐infrared region (SWIR1 in 1550–1760 nm and SWIR2 in 2025–2310 nm) which ranges from 1300 to 2500 nm. The reflectance factors CCRF, HCRF, and DHRF are hereby referred to simply as reflectance factors (RF). For the vascular plant species measured at leaf level, the RF of the leaves' adaxial sides were averaged, and the resulting mean spectrum was used as the representative for that species. All processing of the data was done using R software (R Core Team 2024).

The endmember species used in the spectral unmixing model were selected based on their abundance in the plots so that the species that occurred in fewer than five plots and had < 5% coverage in these plots were excluded from the spectral unmixing analysis. This resulted in six species being used as endmembers in the unmixing model for minerotrophic plots and six species being used as endmembers for the ombrotrophic plots (Table 2). The vegetation plots were classified into two peatland types: minerotrophic (63 plots) and ombrotrophic (61 plots). Correspondingly, the endmember species were categorized as either minerotrophic or ombrotrophic, resulting in five plant species in each category. Litter, which was present in the vegetation plots on both peatland types, was included in the endmember set of both trophic levels.

**TABLE 2:** The species selected as the endmembers in the non‐negative least squares (NNLS) model, as well as their vegetation groups.

Model	Species	Vegetation group
Minerotrophic model	*Comarum palustre*	Vascular plant
	*Filipendula ulmaria*	Vascular plant
	Litter	
	*Menyanthes trifoliata*	Vascular plant
	*Sphagnum angustifolium*	*Sphagnum* moss
	*Sphagnum fallax*	*Sphagnum* moss
Ombrotrophic model	*Calluna vulgaris*	Vascular plant
	Litter	
	*Rubus chamaemorus*	Vascular plant
	*Sphagnum cuspidatum*	*Sphagnum* moss
	*Sphagnum fuscum*	*Sphagnum* moss
	*Sphagnum rubellum*	*Sphagnum* moss

#### 2.1.4 Estimation of the Fractional Cover of Plant Species

We took a near‐nadir viewing photograph of every vegetation plot with a camera (Nikon D3400 with lens AF‐P DX NIKKOR 18–55 mm f/3.5–5.6G VR). The fractional cover of different species present in the plots was systematically estimated from these photographs as follows. The sampling frame that defined the plot area was marked every 10 cm along its sides (Figure 1a,b), and these marks were used to position a digital grid of 100 cells on top of the photograph. Each cell of the grid corresponded to a 10 cm × 10 cm area on the ground. Using in‐house software, we placed a mark in the center of each grid cell and identified and recorded the plant species present at the center of the cell. The fractional covers of each species per trophic level are shown in Table 3.

**TABLE 3:** Fractional cover of the species at the measured peatland sites.

Species, minerotrophic plots	Fractional cover, all plots (%)	Fractional cover, Kurjenrahka (%)	Fractional cover, Tammeluha (%)	Fractional cover, Teijo (%)	Fractional cover, Torronsuo (%)	Fractional cover, Valkmusa (%)
*Betula nana*	1	3	0	0	0	0
*Calla palustris*	2	10	0	0	0	0
*Comarum palustre*	2	0	5	0	1	0
*Filipendula ulmaria*	3	0	7	0	0	0
Litter	31	18	31	40	5	62
*Menyanthes trifoliata*	8	5	0	0	70	0
*Phalaroides arundinacea*	22	0	51	0	0	0
*Ranunculus repens*	0	0	1	0	0	0
*Rubus chamaemorus*	1	3	0	0	0	0
*Salix* sp.	2	0	5	0	0	0
*Sphagnum angustifolium*	7	0	0	54	0	0
*Sphagnum centrale*	0	0	0	0	1	0
*Sphagnum fallax*	18	48	0	6	23	38
*Vaccinium myrtillus*	1	3	0	0	0	0
*Vaccinium uliginosum*	2	8	0	0	0	0
*Vaccinium vitis‐idaea*	0	0	0	0	1	0
*Viola palustris*	0	0	0	0	0	0

Species, ombrotrophic plots	Fractional cover, all plots (%)	Fractional cover, Kurjenrahka (%)	Fractional cover, Luhasoo (%)	Fractional cover, Männikjärv (%)	Fractional cover, Patvinsuo (%)	Fractional cover, Soomaa (%)	Fractional cover, Teijo (%)	Fractional cover, Torronsuo (%)	Fractional cover, Valkmusa (%)
*Betula nana*	0	0	0	0	0	0	0	0	0
*Calluna vulgaris*	19	33	0	23	0	13	30	22	29
*Chamaedaphne calyculata*	0	0	2	0	0	0	0	0	0
Litter	43	20	67	58	34	51	28	26	52
*Rubus chamaemorus*	1	6	0	0	4	0	0	4	0
*Sphagnum angustifolium*	8	7	13	0	37	0	12	3	0
*Sphagnum capillifolium*	1	6	6	0	0	0	0	0	0
*Sphagnum cuspidatum*	3	0	0	9	0	0	10	0	0
*Sphagnum fuscum*	11	26	0	8	25	8	4	20	7
*Sphagnum rubellum*	13	0	11	2	0	25	16	25	12
*Vaccinium myrtillus*	0	0	0	0	0	1	0	0	0
*Vaccinium uliginosum*	0	2	2	0	0	1	0	0	0
*Vaccinium vitis‐idaea*	0	0	0	0	0	1	0	0	0

#### 2.1.5 Simulating Multispectral Data

To compare the potential of hyperspectral to multispectral data in assessing species cover, we resampled the hyperspectral data to multispectral data. The resampling was done using the spectral response functions of the Sentinel‐2C MSI sensor (Copernicus Sentinel‐2 n.d., S2‐SRF version 4.0). The Sentinel‐2 band 10 could not be simulated because of the missing data in the water absorption regions. We also excluded bands 1 and 9 because of their coarser 60 m spatial resolution, which limits their applicability to vegetation monitoring. As a result, the bands used in this study, as well as their central wavelengths, were: B2 (490 nm), B3 (560 nm), B4 (665 nm), B5 (705 nm), B6 (740 nm), B7 (783 nm), B8 (842 nm), B8a (865 nm), B11 (1610 nm), and B12 (2190 nm).

### 2.2 Data Analysis

First, using R software (R Core Team 2024) for all the data analyses, we examined the spectral properties of the vegetation communities (plots) as well as the species‐specific spectra. To analyze how much different wavelength regions explain the variability of the spectral signature of the vegetation plots, we employed a least squares estimation. This was done by fitting a simple linear regression model where the estimated cover fraction of each plant species was the explanatory variable, and the spectral reflectance factor was the response variable. To estimate the goodness of fit, the coefficient of determination (*R* ^2^) was computed. Furthermore, to assess the impact of fractional cover on the model's goodness‐of‐fit, we examined the mean, standard deviation, and maximum values of fractional cover of each endmember species.

To analyze the relationships between spectral signatures and species diversity, we calculated the α‐diversity for each vegetation plot. The α‐diversity, or within‐community diversity, is often expressed with the Shannon‐Wiener Index or the Simpson Index. Both indices express the community's ecological diversity by quantifying the number of species and the distribution of individuals across the species. Therefore, we calculated both the Shannon‐Wiener Index and the Simpson Index to capture potential differences in species richness and evenness, as richness is more strongly reflected by the Shannon‐Wiener Index and evenness by the Simpson Index (Kim et al. 2017; DeJong 1975). We used vegetation fractional cover estimates to calculate the indices for each plot and included all the species present in the vegetation plots in the calculation. The calculation was done using the R package vegan (Oksanen et al. 2024). The resulting index values were found to be normally distributed based on Shapiro–Wilk's normality test.

Then, we used spectral unmixing to assess how the reflectance spectra of the vegetation communities can be used to estimate the fractional cover of individual plant species. Spectral unmixing was performed using nonnegative least squares regression (NNLS), a commonly applied method for spectral unmixing of vegetation (i.e., Canham et al. 2011; Moncholi‐Estornell et al. 2023; Van Wittenberghe et al. 2024). The NNLS was conducted using the R package nnls (Mullen and van Stokkum 2007) to estimate the contributions of the endmembers so that the spectra of vegetation plots were used as the response variables and the endmember spectra were used as the predictors. Here, we used both the hyperspectral and simulated multispectral data as alternative input to the model. The model was then fitted individually for the two trophic levels, using their respective endmember species as the endmember datasets (Table 2). The result was then normalized, and observations of species cover that were 0 were removed. The prediction accuracy of the model was evaluated using the coefficient of determination (*R* ^2^), root mean square error (RMSE) and bias, which were calculated using the R package Metrics (Hamner and Frasco 2018).

Lastly, we performed a partial least squares (PLS) regression to investigate how the spectral signature of a vegetation plot can be used to evaluate the plot's α‐diversity. Since the PLS regression model is especially good at managing collinearity problems in linear models, it was chosen to handle multicollinearity in our data (Wold et al. 1984). We fitted the model using the R package plsRglm (Bertrand and Maumy‐Bertrand 2023), with the model family specified as “gaussian”, which uses normally distributed, continuous data as input, and the link function as “identity.” The models were created separately for both indices for the minerotrophic and ombrotrophic plots. To address model overfitting and determine the optimal number of components, we implemented a 10‐fold cross‐validation. In modeling the Shannon‐Wiener Index, the optimal number of components was determined as five for the minerotrophic model and seven for the ombrotrophic model, whereas for the Simpson Index, it was five and six, respectively. Based on these components, we created the final model to predict the α‐diversity of the vegetation plots. We trained the final model using 70% of the data and used the remaining 30% as test data. Similar to the spectral unmixing model, we used *R* ^2^, RMSE, and bias to evaluate the accuracy of the model.

## 3 Results

### 3.1 Spectral Characteristics of the Vegetation

**FIGURE 2:**
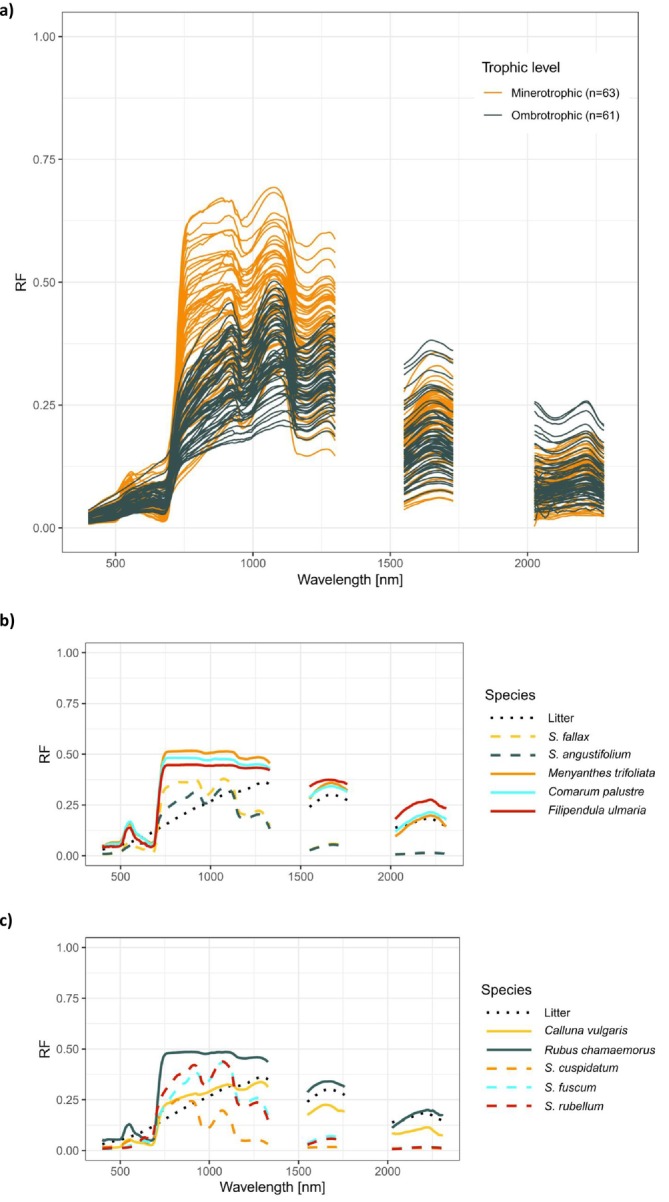
The reflectance factor (RF) of (a) the vegetation plots visualized with their trophic levels, (b) the minerotrophic endmember species and (c) the ombrotrophic endmember species.

The reflectance spectra of the vegetation plots exhibited distinct characteristics between the two trophic levels (Figure 2a). Minerotrophic plots, typically characterized by visually brighter, larger‐leaved vascular plant vegetation, often had a higher reflectance factor in VIS, specifically in the green region (~500–565 nm) compared to ombrotrophic plots (Figure 2a). In the NIR region, the minerotrophic plots bore a visibly higher reflectance factor than the ombrotrophic plots. In the red‐edge region (~670–760 nm), the ombrotrophic plots had a less rapid increase in the reflectance values than the minerotrophic ones (Figure 2a). In the SWIR region, the reflectance spectra of the ombrotrophic plots were, in general, more variable, and on some occasions, had a higher reflectance factor than any of the minerotrophic plots. However, the minerotrophic plots had generally higher reflectance in the SWIR region (Figure 2a). The spectral characteristics of the plant species differed most notably between the plant groups (Figure 2b,c). The herbaceous vascular plants (e.g., *Menyanthes trifoliata, Comarum palustre*) exhibited higher reflectance in the green and the red‐edge regions than the bryophytes (e.g., *Sphagnum angustifolium, Sphagnum fallax*) (Figure 2b). The only shrub species, *Calluna vulgaris* , resembled litter more closely than any other species in both the minerotrophic and ombrotrophic groups (Figure 2c). The properties of the litter spectra were generally typical of dead vegetation, with an evenly increasing reflectance factor in the VIS and NIR regions and relatively high reflectance factor in the SWIR region (Figure 2b,c).

### 3.2 Estimating Species‐Specific Fractional Cover From Hyper‐ and Multispectral Data

**FIGURE 3:**
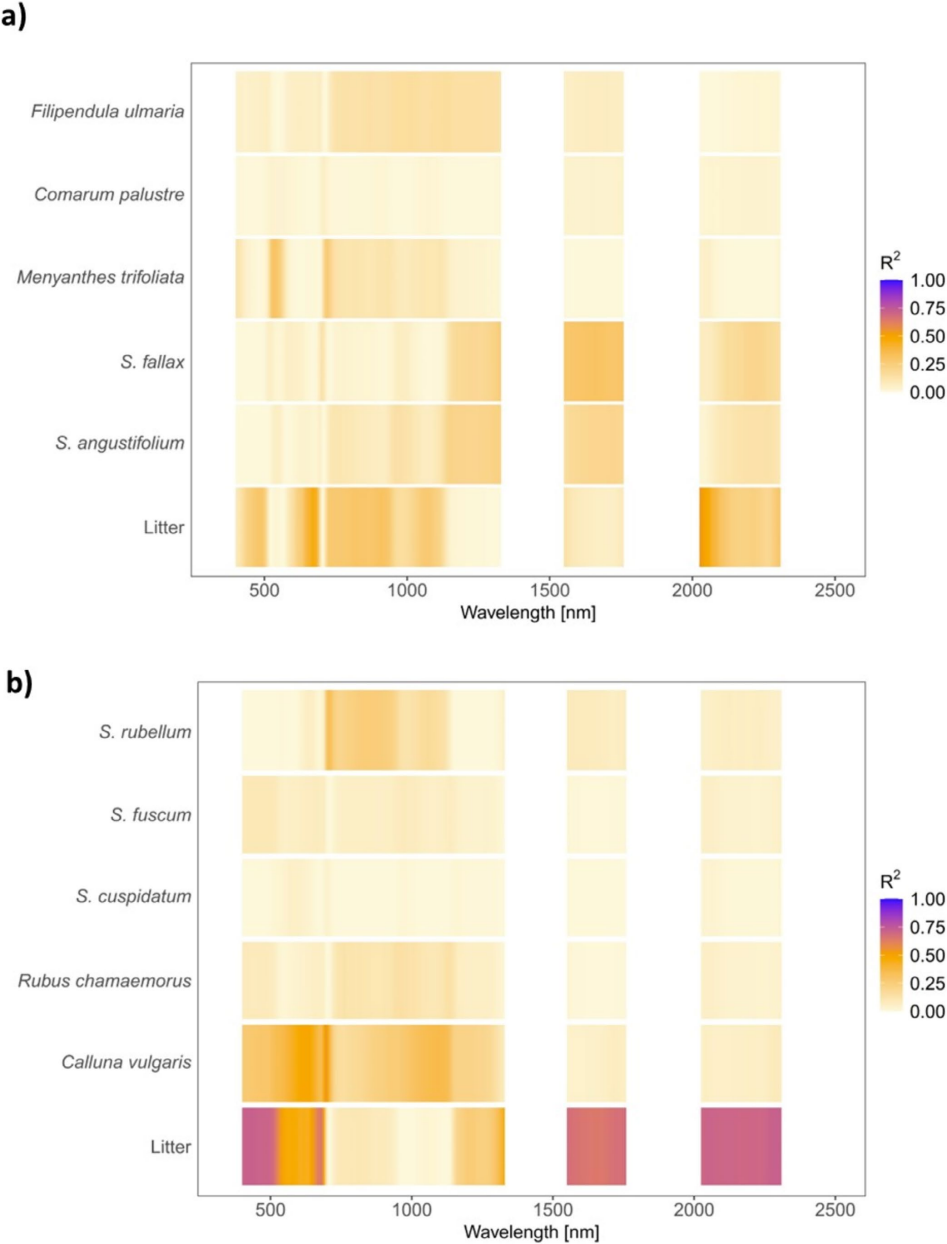
The relationship of the spectral reflectance factor (RF) and the species‐specific fractional cover in the vegetation plots, as depicted by the coefficient of determination (*R* ^2^) of the simple linear regression model explaining the reflectance factor with species fractional cover, for (a) the minerotrophic plant species and (b) the ombrotrophic plant species.

First, we examined results based on hyperspectral data. In the simple linear regression model using the fractional cover to explain the reflectance factor, the fractional cover of the plant species had weak to moderate correlation with the reflectance factor of the vegetation plots across the measured spectrum (Figure 3, Table 4). The coefficient of determination was the highest for litter both in the ombrotrophic plots (*R* ^2^ = 0.72) and the minerotrophic plots (*R* ^2^ = 0.54) (Figure 3, Table 4). For the other species, the relationship was weak (Figure 3, Table 4).

**TABLE 4:** The best coefficient of determination (*R* ^2^) of the different plant species in the simple linear model depicting the relationship of reflectance spectra of the vegetation plots and the species fractional cover, presented in the descending order of the best goodness‐of‐fit together with the mean, maximum, and standard deviation of the estimated fractional cover for each species.

Trophic level	Species	Highest *R* ^2^ of the simple linear model	Mean fractional cover (%)	Maximum fractional cover (%)	Standard deviation of fractional cover (%)
Minerotrophic	Litter	0.54	30	88	22
	*Sphagnum fallax*	0.31	17	86	26
	*Menyanthes trifoliata*	0.31	7	90	20
	*Sphagnum angustifolium*	0.23	6	97	21
	*Filipendula ulmaria*	0.16	3	49	10
	*Comarum palustre*	0.05	2	41	7
Ombrotrophic	Litter	0.72	41	86	21
	*Calluna vulgaris*	0.55	18	80	24
	*Sphagnum rubellum*	0.34	13	86	21
	*Rubus chamaemorus*	0.15	1	12	3
	*Sphagnum fuscum*	0.10	11	62	16
	*Sphagnum cuspidatum*	0.05	3	47	9

**FIGURE 4:**
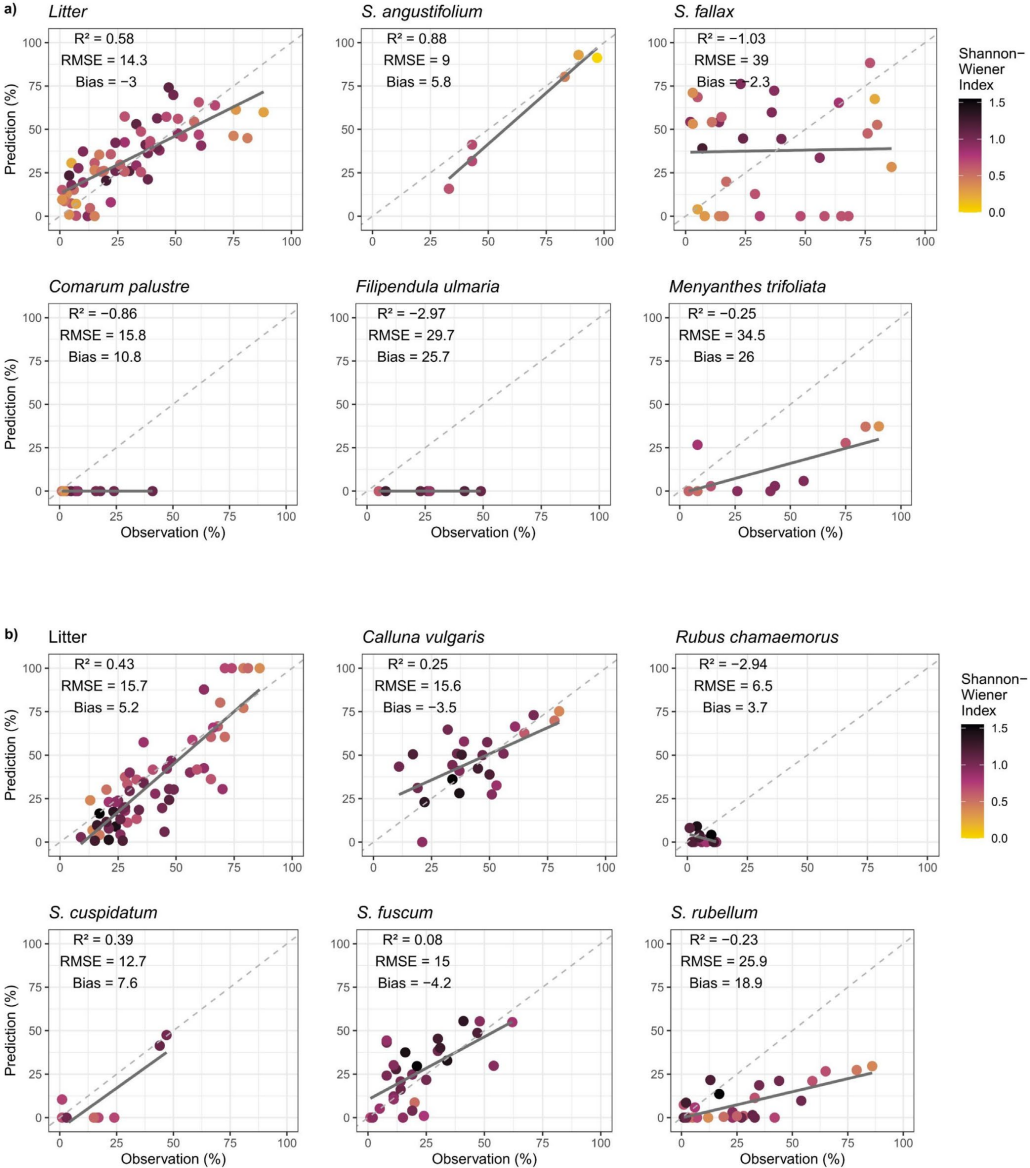
The performance of the non‐negative least squares regression in predicting the fractional covers (%) of endmember species for (a) the minerotrophic plots using the minerotrophic endmembers and (b) the ombrotrophic plots using the ombrotrophic endmembers. The color of the observation indicates the Shannon‐Wiener Index of the vegetation plot.

In the NNLS‐models using hyperspectral data as the input, the fractional cover of the plant species could be predicted with different accuracies. For the minerotrophic model, the best goodness‐of‐fit was achieved with litter (*R* ^2^ = 0.58) and with *Sphagnum angustifolium* (*R* ^2^ = 0.88), which also yielded the lowest RMSE (Figure 4a, Table 5) but was likely impacted by the small sample size. Furthermore, the bias of the different species showed less systemic error in the predictions of these species than in the other species in this model. For *Comarum palustre* and *Filipendula ulmaria* , the model yielded negative R^2^ and high RMSE compared to the range of their observed fractional cover, signifying poor model fit (Figure 4a, Table 5). Additionally, the predicted fractional cover of *Sphagnum fallax* , which had a wide distribution of observed fractional cover, was estimated with poor accuracy (*R* ^2^ = −1.0, RMSE = 39), which deviated from other species with similar distributions of observed fractional covers, such as litter and *Menyanthes trifoliata* (Figure 4a, Table 5). Visual inspection revealed little consistency in the relationship between the α‐diversity of the vegetation plots and the predicted fractional cover of the species (Figure 4a, Table 5).

**TABLE 5:** The coefficient of determination (*R* ^2^), Root Mean Square Error (RMSE) and bias of the non‐negative least squares (NNLS) models that predict the vegetation fractional cover of the two trophic level plots, using hyperspectral (HS) and multispectral (MS) data as the model input.

Species	*R* ^2^ (HS model)	*R* ^2^ (MS model)	RMSE (HS model)	RMSE (MS model)	Bias (HS model)	Bias (MS model)
**Minerotrophic vegetation plots**						
Litter	0.58	0.55	14.3	14.7	−3	5.5
*Sphagnum angustifolium*	0.88	0.11	9	24.1	5.8	21.7
*Sphagnum fallax*	−1.03	−1.19	39	40.6	−2.3	−9.4
*Comarum palustre*	−0.86	−0.86	15.8	15.8	10.8	10.8
*Filipendula ulmaria*	−2.97	−0.04	29.7	15.3	25.7	−0.9
**Ombrotrophic vegetation plots**						
*Menyanthes trifoliata*	−0.25	−0.54	34.5	38.2	26	30.1
Litter	0.43	0.45	15.7	15.5	5.2	5.4
*Calluna vulgaris*	0.25	−0.05	15.6	18.5	−3.5	0.1
*Rubus chamaemorus*	−2.94	−6.53	6.5	8.9	3.7	−2
*Sphagnum cuspidatum*	0.39	0.4	12.7	12.5	7.6	7.9
*Sphagnum fuscum*	0.08	−0.03	15	15.8	−4.2	−4.3
*Sphagnum rubellum*	−0.23	−0.22	25.9	25.8	18.9	18.7

Similarly to the minerotrophic model, the ombrotrophic model with hyperspectral data as the input revealed high variability in the prediction accuracy of the fractional cover of different plant species. The highest, although modest, accuracy was achieved with litter (*R* ^2^ = 0.43). The prediction of *Sphagnum cuspidatum* fractional cover (*R* ^2^ = 0.39) was influenced by two outlier observations and may not indicate robustness in model fit. The prediction accuracy for *Calluna vulgaris* was low (*R* ^2^ = 0.25) and even lower for the rest of the species (Figure 4a, Table 5). However, the low RMSE of some of the species indicated a better model fit; especially litter (RMSE = 15.7), *Calluna vulgaris* (RMSE = 15.6) and *Sphagnum fuscum* (RMSE = 15). The bias for the species was generally low, with the notable exception for *Sphagnum rubellum* (Bias = 18.9), indicating that the model predicts the fractional cover of *Sphagnum rubellum* much higher than what is observed. In a similar way as for the minerotrophic NNLS model with hyperspectral data as the input, visual inspection of the relationship between the α‐diversity and predicted fractional cover of the species revealed no consistent relationship between the two.

Next, we examined results on predicting species‐specific fractional covers based on multispectral data. Compared to the NNLS model with hyperspectral data as the input, the model based on multispectral data showed generally slightly poorer fits for the species with wide distributions of observed fractional covers, such as litter in the minerotrophic model (*R* ^2^ = 0.55, RMSE = 14.7), *Mentyanthes trifoliata* (*R* ^2^ = −0.54, RMSE = 38.2), *Calluna vulgaris* (*R* ^2^ = −0.05, RMSE = 18.5) and *Sphagnum fuscum* (*R* ^2^ = −0.03, RMSE = 15.8) (Table 5). In the minerotrophic model, the fractional cover of litter, *Sphagnum angustifolium* , *Sphagnum fallax* , and *Mentyanthes trifoliata* could be predicted with poorer model fit than in the model based on hyperspectral data, in terms of *R* ^2^, RMSE, and bias (Table 5). *Filipendula ulmaria* , for which the NNLS model failed to produce accurate cover predictions either with hyper‐ or multispectral data as the input, showed higher *R* ^2^, RMSE, and bias with the latter (Table 5). For the ombrotrophic species, the model based on multispectral data showed less accurate model fits for *Calluna vulgaris* (*R* ^2^ = −0.05, RMSE = 18.5) and *Rubus chamaemorus* (*R* ^2^ = −2.94, RMSE = 8.9) in terms of *R* ^2^ and RMSE, and *Sphagnum fuscum* (*R* ^2^ = −0.03, RMSE = 15.8) in terms of R^2^, RMSE, and bias. However, in the ombrotrophic model, litter (*R* ^2^ = 0.45, RMSE = 15.5) and *Sphagnum cuspidatum* (*R* ^2^ = 0.4, RMSE = 12.5) showed slightly better model fits with the multispectral input data in terms of *R* ^2^ and RMSE, but not in terms of bias (Table 5). *S. rubellum* showed marginally better fit when using multispectral data as the input.

### 3.3 Estimating α‐Diversity From Hyperspectral Data

We analyzed the α‐diversity of the plots using vegetation data and found distinct patterns in the diversity indices between the two trophic levels. The Shannon‐Wiener Index varied between 0 and 1.24 in the minerotrophic plots and 0.37–1.53 in the ombrotrophic plots, indicating greater α‐diversity in the plots that were measured in ombrotrophic sites. Similarly, the Simpson Index ranged from 0 to 0.68 for the minerotrophic plots and from 0.21 to 0.77 for the ombrotrophic plots. The different diversity indices indicated that both the species richness and their evenness were greater in the ombrotrophic plots.

**FIGURE 5:**
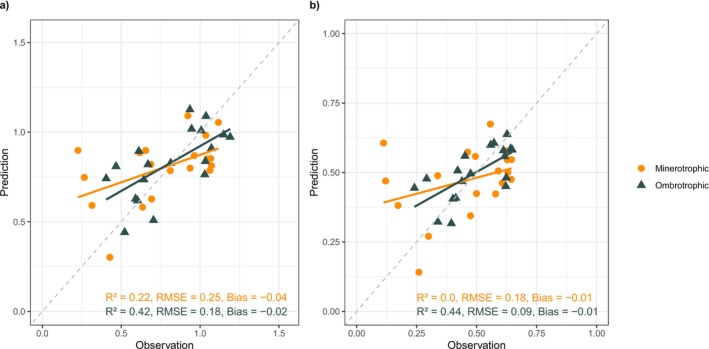
The observed and predicted (a) Shannon‐Wiener Index and (b) Simpson Index of the vegetation plots. The orange dots indicate the models for the minerotrophic plots and the dark triangles indicate the models for the ombrotrophic plots.

Finally, we used PLS regression to predict the α‐diversity for both ombrotrophic and minerotrophic plots based on their hyperspectral reflectance data. Although model performance was modest for both trophic levels, the ombrotrophic plots, characterized by greater variability and a wider distribution of *Sphagnum* mosses and shrubs, showed an overall better model fit compared to the minerotrophic plots, whose vegetation was more heavily dominated by herbaceous species (Table 3). The coefficient of determination of both models was higher in the ombrotrophic plots (*R* ^2^ = 0.42 for Shannon‐Wiener Index, 0.44 for Simpson Index) than in the minerotrophic plots (*R* ^2^ = 0.22 for Shannon‐Wiener Index, 0.0 for Simpson Index); the *R* ^2^ for the Simpson Index was zero, indicating that none of the variation of the Simpson Index's α‐diversity values could be explained by the spectral variation (Figure 5a,b). The RMSE for the Shannon‐Wiener Index models was 0.25 and 0.18 for the minero‐ and ombrotrophic plots, respectively, whereas for the Simpson Index models, they were 0.18 and 0.09 for the minero‐ and ombrotrophic plots. The bias for each model was low, indicating little systematic error.

## 4 Discussion

### 4.1 Spectral Characteristics of the Vegetation

We observed distinct spectral characteristics between the vegetation of the different peatland trophic levels. The spectral signatures of the minerotrophic plots closely resembled the spectral signatures described as typical to vascular plants in previous studies (Girard et al. 2020; Meireles et al. 2020); whereas the spectral signatures we observed in the ombrotrophic plots bore more similarities to what previous literature has described as typical to bryophytes (Bubier et al. 1997; Harris 2008; Lees et al. 2019). Overall, in the VIS and NIR regions, the minerotrophic vegetation plots varied from each other to a greater extent than the ombrotrophic plots; whereas the ombrotrophic plots' RF resembled pure bryophyte spectra more uniformly (Figure 2a). The other notable spectral difference between the trophic levels was the green reflectance peak at ~550 nm, which occurred in most minerotrophic plots but was more diminished in the majority of the ombrotrophic plots (Figure 2a). These differences indicate that a larger part of the ombrotrophic plots was dominated by bryophytes; whereas the minerotrophic plots' vegetation was dominated by broadleaved vascular plant vegetation. In the SWIR regions, the ombrotrophic plots exhibited greater variability in their RF compared to the minerotrophic plots, as they included both the highest reflectance factor and most of the lowest values (Figure 2a). The high reflectance factor was associated with a dry plot, and a low reflectance factor corresponded to a plot with higher soil moisture content. This corresponds with the plots' observed fractional cover, where the ombrotrophic plots had both the highest cover fractions for both litter and bryophytes (Table 3).

### 4.2 Predicting Species‐Specific Fractional Cover of Vegetation Plots

To determine whether hyperspectral or multispectral data offers better predictive performance, and to assess how well species‐level fractional cover can be estimated, we compared the performance of models using both data types in estimating species‐specific vegetation fractional cover. In the model that used multispectral data as input, the fits were moderate to weak, similar to the model that utilized hyperspectral data (Table 5). Although most of the endmembers with a larger sample size and even distribution across the cover percentage range had slightly better model fits using hyperspectral data, the ombrotrophic litter and *Sphagnum cuspidatum* could be predicted with slightly higher accuracy with multispectral data (Table 5). Even if both hyperspectral and multispectral data yielded moderate to low model fits, the result indicates that, for some cover types, the lower‐cost multispectral sensors could provide accuracy comparable to that of hyperspectral data. Previous literature has reported similar findings in mapping ground vegetation attributes in other types of ecosystems: Lu et al. (2019) found comparable performance between hyperspectral and multispectral imaging in predicting leaf chlorophyll content in temperate grassland vegetation. Jarocińska et al. (2022, 2023) reported conflicting results on habitat classification, with one study favoring multispectral data and the other hyperspectral data in temperate wetlands. The variability in our results mirrors the varying outcomes reported earlier, and the limited success in our classification highlights the inherent challenges of species‐level classification in complex peatland vegetation communities.

In estimating the fractional cover of species with spectral unmixing, the models for the two trophic levels yielded notably different accuracies. The highest prediction accuracies were obtained for two endmembers in the minerotrophic model: litter and *Sphagnum angustifolium* . The model's performance was the strongest in predicting litter cover, both with the hyperspectral and multispectral data. This was likely due to its distinct non‐vegetation spectral profile (Figure 2b,c). Furthermore, litter's large sample size and even distribution across the complete cover percentage range likely impacted the model performance. Contrastingly, *Sphagnum angustifolium* had only six observations, which exaggerated the model's prediction accuracy, especially in the model based on hyperspectral data. Using hyperspectral data, a larger number of moderate prediction accuracies were achieved in the ombrotrophic model. This was likely due to the greater abundances in the species fractional cover, for example, for *Calluna vulgaris* , *Sphagnum fuscum* , and *Sphagnum rubellum* (Table 3).

Differences in the prediction accuracy for the trophic levels might also be partially linked to the distinct growing styles of the minerotrophic and ombrotrophic vegetation. The ombrotrophic species with high fractional cover (Table 3) would often grow low or flat, whereas the minerotrophic species with high fractional cover (Table 3) were more typically taller and visually structurally more similar to each other (e.g., the leaves of *Comarum palustre* and *Filipendula ulmaria* resemble each other more closely than the leaves of *Calluna vulgaris* and *Rubus chamaemorus* ). However, the small cover fractions of *Comarum palustre* and *Filipendula ulmaria* likely limited the model's ability to predict their cover fraction. Overall, the results of the model with the hyperspectral data input suggest that assessing peatland vegetation at the species level is more achievable in the ombrotrophic habitats, where the cover fraction of a single species is greater. Moreover, a flatter, less layered growing style of the vegetation community (Table 3) appears to improve the prediction accuracy to some extent (e.g., with *S. fuscum* , which forms a flat ground layer with little vegetation to cover it, versus *Sphagnum fallax* , which typically forms a flat ground layer with a denser cover of tall vascular vegetation growing on top of it). This observation agrees with previous studies, where the bryophyte‐dominated habitats have been found to be more useful in assessing moisture and greenhouse gas emissions than the habitats with more layered vegetation (Burdun et al. 2023; Tucker et al. 2022; Meingast et al. 2014). Furthermore, distinguishing peatland bryophytes from vascular plants has been found to be more feasible than differentiating vascular plant types from each other with drone‐based optical data (Steenvoorden and Limpens 2023).

Our study suggests that, despite the relatively high number of plant species (endmembers), the mapping of species can be achieved in minerotrophic sites with modest accuracy for some endmembers, namely litter, whereas in ombrotrophic sites, accuracy tends to be low but extends across a broader range of species. This observation indicates that while minerotrophic vegetation may allow detection of litter with moderate accuracy, the ombrotrophic taxa, with their often higher cover fractions (Figure 4, Table 3), offer a broader, although much less accurate, species‐level detection. The effect is more pronounced with hyperspectral data but is also evident in the model using multispectral data. Nevertheless, our results indicate that the use of species‐specific spectra appears to be less effective than, for example, using plant functional types in vegetation mapping. Previous research has shown that applying plant functional types in peatland vegetation mapping produces more accurate and more readily generalizable results both with hyper‐ and multispectral data (Salko, Hovi, Burdun, et al. 2024a, 2024b; Pang et al. 2022; Räsänen, Juutinen, et al. 2020; McPartland et al. 2019; Schaepman‐Strub et al. 2009). This suggests that the plant functional type approach is, overall, more effective than attempting to apply species‐specific spectra for predicting vegetation cover in these complex communities.

Finally, in peatlands representing both trophic levels, the fractional cover of litter was predicted with moderate accuracy. This aligns with previous research, which has found litter and other non‐photosynthetic vegetation spectra to be distinguishable with relative ease from photosynthetic vegetation in agricultural environments (Zhang et al. 2025; Pepe et al. 2020; Dennison et al. 2019) and grassland ecosystems (Kowalski et al. 2022). The relative proportion of litter in the vegetation communities varies depending on the time of the growing season, as the amount of annual vascular plants increases towards the peak of the growing season (Wilson et al. 2007; Antala et al. 2022). The ability to monitor litter and abiotic ground components, such as bare soil, based on their spectral reflectance has been utilized to study seasonal dynamics in deciduous forest ecosystems (Wang et al. 2017). Thus, using the peatland‐specific litter spectra, the ability to detect the litter cover fraction could hold potential for improving the monitoring of seasonal vegetation dynamics also in peatland ecosystems.

### 4.3 Predicting α‐Diversity of Vegetation Plots

The α‐diversity of vegetation plots, measured using both the Shannon‐Wiener Index and Simpson Index, was slightly higher in the ombrotrophic plots compared to the minerotrophic ones. Although the minerotrophic plots entailed a larger number of species overall (Table 3), they tended to be distributed across different plots, whereas the ombrotrophic species were more likely to co‐occur within the same plot (Table 3). It is likely that this resulted in somewhat higher α‐diversity within the ombrotrophic plots.

The models utilizing hyperspectral data to predict the α‐diversity of ombrotrophic plots demonstrated a better fit compared to those predicting it for minerotrophic plots. The higher coefficient of determination suggests a stronger association between the spectral signature and the α‐diversity of the ombrotrophic plots; the lower RMSE values indicate that the model predictions are more precise for the ombrotrophic than the minerotrophic plots.

In previous research, α‐diversity has been successfully detected both with hyperspectral and multispectral data based on airborne and satellite images, but these findings are not from peatland ecosystems. Hyperspectral data was used to predict α‐diversity with *R* ^2^ of > 0.7 in temperate grassland ecosystems (Gholizadeh et al. 2020; Wang et al. 2024). Multispectral data yielded similar R^2^ in predicting α‐diversity in Mediterranean forests (Serwah Boakye et al. 2023), and showed advantages compared to hyperspectral data in predicting α‐diversity in mangrove forests (Wang et al. 2022). A laboratory study classifying the α‐diversity of topsoil vegetation communities of a semiarid habitat with hyperspectral data (Blanco‐Sacristán et al. 2019) bore more similar results to ours (*R* ^2^ = 0.47). This may indicate that close‐range sensing of the α‐diversity of small‐scale, highly complex vegetation communities can be more challenging, and potentially less effective, than the remote sensing of the α‐diversity of broader vegetation groups and trees. Furthermore, this highlights the need for further explorations to identify the most reliable and effective methods for detecting peatland α‐diversity.

In some individual minerotrophic plots, the observed α‐diversity was low, but the predicted values were high. This observation was likely impacted by the following: as described earlier, the minerotrophic species were more often distributed across different plots, whereas the ombrotrophic species would often grow together on the same plot. At the same time, the minerotrophic vegetation communities often included a bigger proportion of herbaceous vascular plants than the ombrotrophic ones, which were typically characterized by a more diverse composition of bryophytes, shrubs, and herbaceous plants (Table 3). The herbaceous vascular plant species bore more spectral similarities to each other than the other species (Figure 2b,c), which likely contributed to the poor performance of especially the model predicting the Simpson Index of the minerotrophic plots (Figure 5b). Furthermore, the ombrotrophic plots bore a greater variability and wider distribution range in the fractional cover of *Sphagnum* mosses (Table 3). Although vascular vegetation present on the ombrotrophic plots had a greater spectral variability and distribution range in their fractional cover (Table 3, Figure 2b,c), the bryophyte cover was probably also contributing to the models' performance. The higher spectral variability of the ombrotrophic *Sphagnum* species (Figure 2b,c; Salko et al. 2023a) likely further contributed to the better model performance in ombrotrophic plots compared to minerotrophic ones. Overall, the results show tentative but limited potential of using hyperspectral data to assess the α‐diversity in ombrotrophic habitats, in contrast to minerotrophic habitats, where species exhibit more spectral similarity to each other.

### 4.4 Future Perspectives

Our findings highlight the potential of hyperspectral data to monitor changes in peatland vegetation diversity, especially between the different trophic levels, and to extend these observations across larger landscapes beyond individual peatlands. Our results suggest modest but generally broader potential for species‐diversity detection in ombrotrophic sites than in minerotrophic ones. This result is encouraging for examining ombrotrophic peatland vegetation diversity in general, as the ombrotrophic peatland site types exhibit overall a more limited variability in their vegetation than minerotrophic ones (Laine and Vasander 1996; Rydin and Jeglum 2006). As such, the ombrotrophic species studied here represent their habitats more broadly than the studied minerotrophic species, making our findings more readily generalizable across ombrotrophic peatland environments. Our results also suggest that monitoring litter fraction in peatlands may be feasible with data from new and forthcoming hyperspectral satellite missions. The spectral unmixing showed promising results in detecting the fractional cover of litter from the peatland vegetation communities. Droughts during the growing season have been observed to result in an increase in litter abundance in peatlands, as vascular plants start to wither (McPartland et al. 2018). Thus, the ability to monitor changes in the relative amount of litter throughout the growing season could enhance the understanding of the sites' seasonal dynamics, which may be shifting in response to climate change (Rastogi et al. 2019). It is reasonable that litter, as non‐photosynthetic vegetation, is spectrally more easily distinguishable from photosynthetically active living vegetation. However, retrieving information solely on litter cover may overlook other important aspects of the dynamics of peatland vegetation communities. The poor success in modeling the fractional cover of different plant species suggests that broader classification strategies, such as plant functional types, may be more efficient for retrieving information on the vegetation cover of these plant communities (Salko, Hovi, Burdun, et al. 2024b).

Furthermore, the spectral data presented in this study can contribute to not only the development of remote sensing methods for peatland vegetation but also to the scientific understanding of the shortwave radiation regime (albedo) of peatland vegetation and its connections to vegetation‐climate feedback mechanisms via radiative transfer modeling of peatlands. Ombrotrophic vegetation is replacing minerotrophic vegetation at an accelerating rate, which has been associated with the changing climate conditions (Kolari et al. 2022; Kuuri‐Riutta et al. 2024; Robitaille et al. 2021). Based on our results, the albedo—or the broadband “reflectivity” of the vegetation integrated over the optical domain—of minerotrophic peatlands can be expected to be higher than that of ombrotrophic peatlands (Figure 2a). In the future, lower albedo may create positive feedback loops that influence the local climate and also amplify climate change.

## 5 Conclusions

We analyzed the potential of hyper‐ and multispectral data in assessing the fractional cover of plant species and α‐diversity of boreal peatland vegetation communities. We demonstrated the spectral variation between the two trophic levels and identified the most informative spectral regions in relation to species fractional cover. The most important spectral regions varied between different species, but were primarily located in the green, red‐edge, and SWIR regions. We showed that the spectral libraries with pure species‐specific spectra can be used to predict the fractional cover of the species to a very limited extent, and that predicting the fractional cover of litter yielded modest accuracies on both trophic levels (for the models using hyperspectral data as the input, *R* ^2^ = 0.58 and *R* ^2^ = 0.43 in minero‐ and ombrotrophic plots, respectively, and *R* ^2^ = 0.55 and *R* ^2^ = 0.45 for the models using multispectral data as the input). Finally, our analyses revealed that in ombrotrophic plots, the α‐diversity of peatland vegetation communities can be predicted with low to moderate accuracy (*R* ^2^ = 0.42 and 0.44 with Shannon‐Wiener and Simpson index, respectively) using the spectral signature of the vegetation community, which is reasonable given the complexity of the vegetation communities. The new and upcoming hyperspectral satellite missions, such as EnMAP, PRISMA, and CHIME, will provide complete coverage of the shortwave spectrum from around 400 to 2400 nm with high spectral resolution. Thus, methods for large‐scale monitoring of the vegetation conditions in boreal peatlands will also advance our real‐time understanding of their carbon balance. The insight into the spectral properties of boreal peatland vegetation provided in our study can be applied to the development of large‐scale monitoring of vegetation conditions and diversity of these vital ecosystems.

## Author Contributions

**Sini‐Selina Salko:** conceptualization (equal), data curation (equal), formal analysis (lead), investigation (equal), methodology (lead), software (lead), visualization (lead), writing – original draft (lead). **Aarne Hovi:** data curation (equal), writing – review and editing (equal). **Iuliia Burdun:** data curation (equal), investigation (equal), writing – review and editing (equal). **Jussi Juola:** writing – review and editing (equal). **Susanna Karlqvist:** data curation (equal), writing – review and editing (equal). **Miina Rautiainen:** conceptualization (equal), funding acquisition (lead), project administration (lead), supervision (lead), writing – review and editing (equal).

## Conflicts of Interest

The authors declare no conflicts of interest.

## Data Availability

Data are available at https://doi.org/10.17632/3866tj3w8v.1 (Salko, Hovi, Burdun, et al. 2024a, spectral library of the vegetation plots), https://doi.org/10.17632/wm5fcxdmzd.3 (Salko et al. 2023a, spectral library of the *Sphagnum* mosses) and https://doi.org/10.17632/kg7627wgp6.1 (Salko, Hovi, Juola, and Rautiainen 2024, spectral library of the vascular plants and litter).
